# Reversal of fibrosis in patients with nonalcoholic steatohepatosis after gastric bypass surgery

**DOI:** 10.1186/s40608-017-0168-y

**Published:** 2017-09-12

**Authors:** Brian M. Parker, Jiang Wu, Jing You, David S. Barnes, Lisa Yerian, John P. Kirwan, Philip R. Schauer, Daniel I. Sessler

**Affiliations:** 1 Department of General Anesthesiology, Anesthesiology Institute, Cleveland Clinic, Cleveland, OH USA; 2 Department of Anesthesiology & Pain Medicine, University of Washington Medical Center, Seattle, WA USA; 3 Departments of Quantitative Health Sciences and OUTCOMES RESEARCH, Anesthesiology Institute, Cleveland Clinic, Cleveland, OH USA; 4 Department of Gastroenterology and Hepatology, Cleveland Clinic, Cleveland, OH USA; 5 Department of Anatomic Pathology, Cleveland Clinic, Cleveland, OH USA; 6 Department of Pathobiology, Cleveland Clinic, Cleveland, OH USA; 7 Department of General Surgery, Cleveland Clinic, Cleveland, OH USA; 8 Department of Outcomes Research, Anesthesiology Institute, Cleveland Clinic, Cleveland, OH USA; 9 Department of Anesthesiology, Allegheny Health Network, Pittsburgh, PA USA

**Keywords:** Anesthesia, Liver, Roux-en-Y gastric bypass, Fatty, Non-alcoholic steatohepatitis, Fibrosis

## Abstract

**Background:**

Roux-en-Y gastric bypass (RYGB) improves the pathophysiology that contributes to obesity-related nonalcoholic steatohepatitis (NASH).

Whether obesity-related fibrosis improves is unclear. We hypothesized that RYGB reverses NASH and fibrosis, and indocyanine green (ICG) clearance provides a sensitive measure for detecting asymptomatic fatty liver disease.

**Methods:**

One hundred six obese adults scheduled for RYGB had preoperative liver function assessed using standard tests and ICG clearance and core liver biopsies obtained during RYGB. Once patients lost 60% of their preoperative weight or weight loss plateaued, liver function was reassessed. Repeat liver biopsies were obtained on patients with NASH at the time of RYGB.

**Results:**

RYGB improved steatosis, lobular inflammation, hepatocyte ballooning and fibrosis. Serum albumin, AST, and ALT decreased the most in patients with NASH and NASH plus fibrosis. Twenty seven (26%) patients had normal baseline liver histology and 45 (43%) had NASH or NASH plus fibrosis. Nine of 13 patients with substantial fatty liver had normalized histology after weight loss, while severity of disease in the rest had stabilized or was reduced. Mean ICG clearance in patients with normal/mild fatty liver disease and those with histological fatty livers did not differ significantly.

**Conclusions:**

RYGB surgery reverses NASH and liver fibrosis. Underlying mechanisms that facilitate improvement remain unclear.

## Background

Over one-third of all Americans are obese, as determined by a body mass index (BMI) of ≥30 kg/m^2^. The prevalence of morbid obesity (BMI ≥40 kg/m^2^) has increased by a factor of 3.5 in the last 30 years from 1.4% in 1980 to 6.4% in 2012 [1].

Morbid obesity is strongly associated with nonalcoholic fatty liver disease (NAFLD), which is now among the most common worldwide causes of chronic liver disease [2]. Obesity-related degradation of hepatic microscopic architecture is well recognized and ranges in severity from steatosis to nonalcoholic steatohepatitis (NASH) to fibrosis and finally to cirrhosis [3].

Currently, the best treatment of NAFLD and NASH is weight reduction [4, 5]. Surgery, including laparoscopic Roux-en-Y gastric bypass (RYGB), offers the best prognosis for substantial and sustained loss [6, 7].

Weight-loss surgery improves the underlying pathophysiology that contributes to development of obesity-related NASH [8, 9], but whether obesity-related fibrosis improves following RYGB is less clear. Our primary aim was to assess whether RYGB surgery would induce improvements in liver function and histology once significant weight loss had occurred post operation. Specifically, we tested the hypotheses that liver disease observed at the time of surgery is reversible with weight loss, as evidenced by improvements in the standard biochemical tests, indocyanine green (ICG) clearance, and especially histology. Secondarily, we evaluated the diagnostic utility of ICG clearance relative to biochemical testing for detecting asymptomatic, but nonetheless clinically important, fatty liver diseases before and after RYGB.

## Methods

Adults with class II and III obesity, scheduled for RYGB surgery were enrolled in this prospective observational study (NCT00701376). We included patients who had a BMI ≥ 40 kg/m^2^ or >35 kg/m^2^ with obesity related co-morbidities; who had failed non-surgical treatment for morbid obesity; and were scheduled for standardized laparoscopic RYGB surgery.

We excluded patients who had forms of liver disease (such as chronic viral hepatitis, autoimmune hepatitis) unrelated to obesity, evidence of end-stage liver disease including portal hypertension, ascites, and coagulopathy; or known iodine sensitivity or allergy. Participants were verbally briefed about the study and signed informed consent documents approved by the Cleveland Clinic Institutional Review Board.

### Protocol

Preoperative biochemical testing was obtained including aspartate aminotransferase (AST), alanine transferase (ALT), alkaline phosphatase (ALK), total bilirubin, albumin and prothrombin time (PT). Serum lipid profiles and HbA1c values were medially optimized, and patients were asked to refrain from alcohol use for several preoperative days. Potentially hepatotoxic medications were discontinued. Laparoscopic RYGB surgery was performed as previously described [10]. Anesthesia was standardized, and consisted of a volatile gas, an intermediate-acting, non-depolarizing neuromuscular blocker, and intravenous opioids.

One or two core liver tissue samples were extracted from the right lobe under direct visualization with a 14-gauge 15-cm Tru-Cut (Allegiance Healthcare Corp., McGaw Park, IL) biopsy needle. Liver biopsy specimens were fixed in formalin, embedded in paraffin, and appropriately stained for evaluation by the hepatopathologist.

Once patients lost 60% of their preoperative excess weight or weight loss had plateaued, both liver function and histology were reassessed. Follow-up biochemical testing was obtained including AST, ALT, ALK, total bilirubin, albumin and PT. Continued optimization of each patient’s serum lipid profiles and HbA1c values, as well as a required abstinence form alcohol use was on-going.

Repeat percutaneous ultrasound-guided liver biopsies were offered to patients who had stable weight loss and were found to have clinically important liver damage as determined by liver biopsy at the time of RYGB. Based on prior work, we considered NAFLD activity scores (NAS defined below) exceeding three to indicate clinically important liver damage. In this sub-set of patients who qualified and agreed, one or two percutaneous core needle liver biopsies were obtained from the right lobe of the liver. Specimens were prepared and evaluated just as they were preoperatively by a hepatopathologist.

### Measurements

Demographic and morphometric characteristics were recorded, along with the duration of morbid obesity. Additional patient information including a history of viral hepatitis, alcohol or illicit drug use, hyperlipidemia, diabetes mellitus, and cardiovascular disease including hypertension were also collected.

Standard preoperative biochemical testing included AST, ALT, ALK, total bilirubin, albumin, and PT. Immunoassays were used to evaluate hepatitis B surface antigen (HbsAg) and hepatitis C virus (HCV).

Liver function was assessed by measuring ICG clearance using the DDG-2001 Analyzer (Nihon Koden Corporation, Patient Monitoring Systems Division, Tokyo, Japan) [11]. The test was performed in unanesthetized subjects within 3 weeks before RYGB. A 0.5 mg/kg IV bolus of ICG was given within 3 sec via an 18-gauge IV catheter. The DDG-2001 Analyzer constructs a dye-densitogram (graphical representation) of serum ICG clearance using a non-invasive optical pulse-spectrometry finger or nasal probe. This graph of ICG clearance is actually a decay curve with the slope designated as *k*. Thus, *k* represents the rate of disappearance of ICG from the blood as the liver exclusively extracts it. Therefore, the smaller the *k* value the lower the rate of ICG clearance from the blood. The analyzer determines the concentration of ICG (mg/L) by comparing the infrared absorption spectra of ICG at both 805 nm and 940 nm, similar to the methods used in pulse oximetry.

The liver was visually inspected during the RYGB before liver biopsy. Hepatomegaly was defined by the lower edge of the liver being below the gastric margin. Fat speckling or fat infiltration was classified as present or absent.

Liver biopsy specimens were characterized as having normal anatomy, steatosis steatohepatitis, or fibrosis. The NAFLD activity score (NAS) from the NASH clinical Clinic Research Network is the unweighted sum of scores for steatosis, lobular inflammation, and ballooning hepatocyte degeneration, and ranges from zero to eight points. The histological reporting for grading steatosis was based on a scale of 0 to 3, with 0 being no steatosis (<5%), 1 being mild steatosis (involving 5–33% of the biopsy specimen), 2 being moderate steatosis (involving 34–66% of the specimen), and 3 being severe (involving >66%). Lobular inflammation was similarly scored by number of foci per 200× magnification field (0 no foci: 1 < 2 foci: 2, 2–4 foci; 3, >4 foci). Ballooning hepatocyte degeneration was scored as 0 (absent), 1 (few, difficult to identify), 2 (many, easily identified) as seen in Table 1 from Kleiner et al. [3].

**Table 1:** Demographics and preoperative characteristics (*N* = 106)

Variables	Summary statistics
Age, yrs	46 ± 11
Gender^a^ (Male), No. (%)	31 (31)
Race^a^ (Caucasian), No. (%)	90 (89)
Body mass index - kg/m^2^	48 ± 8
Obesity level, No. (%)	
Morbid Obesity	93 (88)
Severe Obesity	11 (10)
Duration of obesity^b^, yrs	26 ± 12

Data presented as means ± SDs or number (percent) of patients

^a^5 and ^b^2 patients with missing values

Fibrosis present in biopsy specimens were staged from 0 to 4, with 0 representing no fibrosis, Stage 1 represents mild (stage 1a), Stage 2 represents portal/periportal and perisinusoidal fibrosis, Stage 3 represents bridging fibrosis (without regenerative nodules). Finally, Stage 4 depicts cirrhosis as seen in Table 1 from Kleiner et al. [3].

### Data analysis

#### Primary analyses

Changes from before to after RYGB for each of the biochemical liver function tests (including AST, ALT, ALK, total bilirubin, albumin, and PT), ICG clearance and histological measures (including NAS steatosis, NAS lobular inflammation, NAS hepatocyte balloon, and fibrosis) were evaluated with paired *t* and Wilcoxon signed rank tests.

We estimated the correlation between changes from baseline in biochemical liver function tests, non-invasive ICG clearance tests, and histological measures using Pearson correlation coefficient or Spearman rank-order correlation, as appropriate.

Bonferoni correction was used to adjust for testing multiple analyses, thus the significance criterion for each of the two primary analyses was *P* < 0.025. Further adjustment was used for multiple comparisons conducted for each analysis; thus 99.8 and 99.83% confidence intervals (CI) were reported for mean change from pre- to post-RYGB n each of the tests and correlations between changes, respectively.

#### Secondary analyses

We grouped patients into one of the following four liver disease categories based on their histological measures prior to surgery. (1) normal liver defined as steatosis <5%, (2) nonalcoholic fatty liver (NAFL) defined as steatosis >5% without any ballooning cells and lobular inflammation <2 foci per 200× magnification field, (3) nonalcoholic steatohepatitis (NASH) defined as steatosis >5% with at least a few balloon cells and lobular inflammation >2 foci per 200× magnification field and no fibrosis or stage 1 or 2 fibrosis, and (4) NASH and fibrosis defined as steatosis >5% with at least a few balloon cells and lobular inflammation >2 foci per 200× magnification field and stage 3 or 4 fibrosis.

We assessed the diagnostic accuracy of either biochemical liver function tests or non-invasive ICG clearance tests at pre-RYGB in relation to the gold standard histological results for clinically asymptomatic but significant fatty liver, including NASH and NASH plus fibrosis. Diagnostic accuracy for each test was assessed by the area (AUC) under the receiver operating characteristic (ROC) curve, which is a widely accepted measure of diagnostic accuracy ranging from 0.50 (chance) to 1.0 (perfect prediction). For each test the significance level was 0.006 (i.e., 0.05/8; Bonferroni correction). Additionally, we built a multivariable logistic regression model to predict significant fatty liver at the pre-RYGB time point from all the available liver function tests and the ICG *k* clearance test. The corresponding AUC was estimated along with its confidence interval. We selected the best observed cutpoint based on jointly maximizing sensitivity and specificity. The corresponding sensitivity, specifically, positive predictive value (PPV), negative predictive value (NPV), as well as prediction accuracy was estimated, along with 95% confidence intervals.

Finally, we evaluated the correlation between ICG *k* clearance values and the total NAS score and liver disease category using the Spearman correlation along with their 97.5% confidence interval.

SAS statistical software 9.3, Carey, NC was used for all analyses. Results are presented as numbers (%), mean ± SDs, or means (specified confidence interval).

## Results

One-hundred-six bariatric surgical patients were enrolled; 105 patients ultimately had RYGB. Demographic and morphometric characteristics are presented in Table 1. A subset of the 37 patients with histologically diagnosed NASH was re-evaluated once patients lost 60% of their preoperative excess weight or weight loss plateaued after surgery.

We performed biochemical liver function tests on 25 of these patients and repeat liver biopsies on 15. All of the statistical analyses were based on complete data.

The average patient follow-up period was 487 ± 86 days with an observed mean decrease in weight of 42 ± 16 kg and a decrease in BMI of 15.6 ± 6.1 kg/m^2^. There was a statistically significant but clinically unimportant reduction in serum albumin concentrations after RYGB of 4.4 versus 4.2 mg/dl (*P* < 0.001). Liver function tests and ICG *k* clearance values did not otherwise differ significantly (Table 2).

**Table 2:** Primary analysis 1 – Estimated mean change from pre- to post-RYGB in each biochemical liver function test, non-invasive ICG clearance test, and histological measures

					Mean Difference	
Variable		N	Pre-RYG	Post-RYGB	(99.8% CI)†	*P*-value†
AST, U/L	25	29.9 ± 9.3	23.6 ± 7.9	−6.32 (−15.6, 2.94)		0.03
ALT, U/L	25	31 [26, 38]	20 [17, 34]	−7.56 (−22.0, 6.83)		0.08
ALK, U/L	25	78.2 ± 19.1	84.0 ± 14.9	5.84 (−7.55, 19.2)		0.14
Total bilirubin, mg/dL	25	0.5 [0.4,	0.4 [0.4,	0.01 (−0.15, 0.17)		0.79
Albumin, g/dL	25	4.4 ± 0.3	4.2 ± 0.3	−0.20 (−0.38, −0.02)		<0.001
PT, second	25	11.3 ± 0.6	11.1 ± 0.5	−0.23 (−0.68, 0.23)		0.10
PTT, second	6	29.8 ± 1.6	28.0 ± 0.3	−1.72 (−8.68, 5.25)		0.21
ICG K value	19	0.21 ± 0.04	0.22 ± 0.06	0.01 (−0.04, 0.06)		0.63
NAS steatosis, No. (%)	15			–		0.002
< 5%		2 (13)	11 (73)			
5–33%		6 (40)	3 (20)			
34–66%		5 (33)	1 (7)			
> 66%		2 (13)	0 (0)			
NAS lobular inflammation, No. (%)	15			–		0.04
No foci		3 (20)	10 (67)			
< 2 foci / 200×		8 (53)	4 (27)			
2–4 foci / 200×		3 (20)	1 (7			
> 4 foci / 200×		1 (7)	0 (0)			
NAS Hepatocyte Balloon, No. (%)	15					0.001
Absent	3 (20)		12 (80)			
Few Balloon Cells	10 (67)		3 (20)			
Many Cells	2 (13)		0 (0)			
Fibrosis, No. (%)	15			–		0.005
None		0 (0)	6 (40)			
Perisinusoidal or periportal		0 (0)	0 (0)			
1A - Mild, zone 3, perisinusoidal		5 (33)	0 (0)			
1B - Moderate, zone 3, perisinusoidal		1 (7)	1 (7)			
1C - Portal/periportal		1 (7)	5 (33)			
2 - Perisinusoidal and portal/periportal		5 (33)	2 (13)			
3 - Bridging fibrosis		3 (20)	1 (7)			
4 - Cirrhosis		0 (0)	0 (0)			

*AST* Aspartate transaminase, *ALT* Alanine transaminase, *ALK* alkaline phosphatase, *ICG* Indocyanine green, *NAS* Nonalcoholic steatohepatitis, *PT* Prothrombin time, *PTT* Partial Thromboplastin Time

†*P*-value for testing the null hypothesis that difference is zero using the paired t-test or Wilcoxon signed rank test, as appropriate. The significance criterion was *P* < 0.002 (i.e., 0.025 / 12; Bonferroni correction)

At the time of RYGB surgery, 27 (26%) patients had normal liver histology; 41 (39%) patients had nonalcoholic fatty liver; 26 (25%) patients had NASH without fibrosis or with stage 1 or 2 fibrosis; and 11 (10%) patients had NASH with stage 3 or 4 fibrosis (Table 3).

**Table 3:** Liver disease category based on liver biopsy at pre- and post-RYGB time points

Liver disease category	Definition	Pre-RYGB	Post-RYGB
		(*N* = 105)	(*N* = 15)
		No. (%)	No. (%)
Normal Liver	Steatosis <5%	27 (26)	11 (73)
	Steatosis >5% without any ballooning		
NAFLD	Cells and lobular inflammation <2 foci per 200 x field	41 (39)	2 (13)
NASH	Steatosis >5% with at least a few balloon cells, lobular inflammation >2 foci per 200 x field	26 (25)	1 (7)
NASH + Fibrosis	Steatosis >5% with at least a few balloon cells, lobular inflammation >2 foci per 200 x field and stage 3 or 4 fibrosis	11 (10)	1 (7)

*NAFLD* nonalcoholic fatty liver disease, *NASH* nonalcoholic steatohepatitis *RYGB* Roux-en-Y gastric bypass

Of the 15 patients with pre- and post-RYGB surgery biopsies, 9/13 patients with substantial fatty liver disease had normalized histological features after weight loss, while the severity of fatty liver disease in the remainder of patients had either stabilized or reduced. Specifically, we observed improvement in steatosis (12/13 patients normalized with reduced severity in the remainder), decreased lobular inflammation (9/12 patients normalized with reduced severity in the remainder), reduced incidence of hepatocyte ballooning (11/12 patients normalized with reduced severity in the remainder), and fibrosis regression (12/15 patients with fibrosis normalized with reduced severity in the remainder (Table 4).

**Table 4:** Liver Biopsy test components for patients who had biopsies both before and after RYGB (*N* = 15)

	Pre-Op RYGB					Post-OP RYGB				
	Lobular		Hepatocyte			Lobular		Hepatocyte		
Pt #	Steatosis	Inflammation	Balloon	Fibrosis^a^	Overall^b^	Steatosis	Inflammation	Balloon	Fibrosis^a^	Overall^b^
26	<5%	No foci	None	3	Normal	<5%	No foci	None	1C	Normal
29	<5%	No foci	None	2	Normal	<5%	No foci	None	2	Normal
38	5–33%	<2 foci/200×	None	1C	NAFL	<5%	No foci	None	0	Normal
4	>66%	<2 foci/200×	Many cells/ prominent ballooning	3	NASH w Fibrosis	5–33%	2–4 foci/200×	Few Balloon Cells	3	NASH w Fibrosis
20	5–33%	<2 foci/200×	Few balloon cells	1B	NASH	<5%	No foci	None	0	Normal
27	>33–66%	<2 foci/200×	Few balloon cells	2	NASH	<5%	No foci	None	1C	Normal
33	>33–66%	<2 foci/200×	Many cells/ prominent ballooning	3	NASH w Fibrosis	<5%	<2 foci/200×	Few Balloon Cells	2	Normal
44	>66%	2–4 foci/200×	Few balloon cells	1A	NASH	<5%	No foci	None	0	Normal
47	5–33%	<2 foci/200×	Few balloon cells	2	NASH	<5%	No foci	None	1C	Normal
50	5–33%	2–4 foci/200×	Few balloon cells	2	NASH	<5%	No foci	None	1C	Normal
60	33–66%	2–4 foci/200×	Few balloon cells	1A	NASH	5–33%	<2 foci/200×	None	1C	NAFL
72	5–33%	No foci	Few balloon cells	2	NASH	>33–66%	<2 foci/200×	Few Balloon Cells	1B	NASH
79	>33–66%	<2 foci/200×	Few balloon cells	1A	NASH	5–33%	<2 foci/200×	None	0	NAFL
83	>33–66%	>4 foci/200×	Few balloon cells	1A	NASH	<5%	No foci	None	0	Normal
102	5–33%	<2 foci/200×	Few balloon cells	1A	NASH	<5%	No foci	None	0	Normal

*NAFL* Nonalcoholic fatty liver, *NASH* Nonalcoholic Steatohepatitis, *RYGB* Roux-en-Y gastric bypass

^a^Fibrosis score: 0 – None; 1 - Perisinusoidal or periportal; 1A - Mild, zone 3, perisunsoidal; 1B - Moderate, zone 3, perisinusoidal; 1C - Portal/periportal; 2 -_28_Perisinusoidal and portal/periportal; 3 - Bridging fibrosis; and 4 – Cirrhosis

^b^We grouped patients into one of the following four liver disease categories based on their histological measures prior to surgery: (1) normal liver defined as steatosis <5%, (2) nonalcoholic fatty liver (NAFL) defined as steatosis >5% without any ballooning cells and lobular inflammation <2 foci per 200 X magnification field, (3) nonalcoholic steatohepatitis (NASH) defined as steatosis >5% with at least a few balloon cells and lobular inflammation >2 foci per 200 X magnification field and no fibrosis or stage 1 or 2 fibrosis, and (4) NASH and fibrosis defined as steatosis >5% with at least a few balloon cells and lobular inflammation >2 foci per 200 X magnification field and stage 3 or 4 fibrosis

All estimated correlations between the changes seen pre- and post-RYGB for biochemical liver function tests, non-invasive ICG *k* clearance, and histological measures ranged from 0.01 (between change in ALT and change in ICG *k* clearance value) to 0.54 (between change in total bilirubin and change in fibrosis). None of these correlations differed significantly from zero (Table 5).

**Table 5:** Primary analysis 2 - Estimated correlations between change from pre- to post- LGBS in each of biochemical liver function tests, non-invasive ICG clearance

Change from pre- to post- LGBS in each of the following test		Pearson correlation		
		Number	(99.83% CI)^†^	*P*-value^†^
ICG K value	AST	19	−0.04 (−0.68, 0.63)	0.88
	ALT	19	0.01 (−0.65, 0.66)	0.98
	Alkaline phosphatase	19	−0.09 (−0.71, 0.60)	0.70
	Total bilirubin	19	0.07 (−0.61, 0.70)	0.76
	Albumin	19	0.30 (−0.44, 0.80)	0.21
	PT	17	−0.24 (−0.80, 0.53)	0.35
	PTT	5	−0.41 (−0.99, 0.95)	0.49
		Spearman correlation		
Fibrosis	AST	12	−0.23 (−1.00, 0.72)	0.47
	ALT	12	0.28 (−0.67, 1.00)	0.38
	Alkaline phosphatase	12	0.33 (−0.61, 1.00)	0.29
	Total bilirubin	12	−0.54 (−1.00, 0.41)	0.07
	Albumin	12	0.03 (−0.91, 0.98)	0.92
	PT	13	−0.35 (−1.00, 0.55)	0.24
	PTT	4	0.45 (−1.00. 1.00)	0.55
	ICG K value	8	0.38 (−0.80, 1.00)	0.35

*AST* Aspartate transaminase, *ALT* Alanine transaminase, *ICG* Indocyanine green, *NAS* Non-alcoholic steatohepatitis, *PT* Prothrombin time, *PTT* Partial Thromboplastin Time

^†^ *P*-value for testing the null hypothesis that correlation is zero. The significance criterion was *P* < 0.0017 (i.e.,0.025/15; Bonferroni correction)

**Fig. 1:**
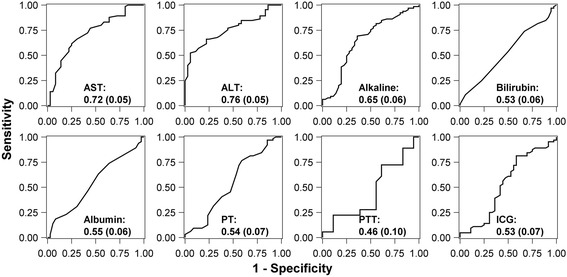
Receiver operating characteristic curves with area under the curve and standard error in parentheses. Displayed are the biochemical liver function tests and non- invasive ICG clearance test at pre-RYGB predicting significant fatty liver disease (nonalcoholic steatohepatitis). ICG = indocyanine green, RYGB = Roux-en-Y gastric bypass

We also observed that preoperative AST (*P* = 0.001) and ALT (*P* < 0.001) were univariably significant predictors of clinically significant fatty livers after RYGB. Both tests had moderate predictive ability of more advanced stages of fatty liver with an AUC of 0.72 (99.4% Cl 0.61 0.82) for AST and 0.76 (0.66, 0.85) for ALT, respectively. Other biochemical liver function tests and ICG *k* clearance values were not significant predictors of NASH (Table 6 and Fig. 1).

**Table 6:** Secondary analysis - Logistic regression and diagnostic accuracy results of biochemical liver function tests and non-invasive ICG clearance test at pre-RYGB for predicting significant fatty liver^a^ at pre-RYGB time point

Test At pre-RYGB	Odds ratio			AUC^b^
	Number	(99.4% CI)	*P* ^†^	(99.4% CI)
AST, U/L	101	1.09 (1.01, 1.17)	0.001	0.72 (0.61, 0.82)
ALT, U/L	101	1.07 (1.01, 1.12)	<0.001	0.76 (0.66, 0.85)
ALK, U/L	101	0.98 (0.95, 1.01)	0.03	0.65 (0.53, 0.77)
Total bilirubin, mg/dL	101	1.20 (0.32, 4.48)	0.70	0.53 (0.41, 0.64)
PT, second	98	1.57 (0.57, 4.34)	0.22	0.54 (0.41, 0.67)
PTT, second	36	0.96 (0.83, 1.10)	0.37	0.46 (0.26, 0.66)
ICG *k* value	100	0.86 (0.27, 2.72)	0.71	0.53 (0.41, 0.66)

*AST* Aspartate transaminase, *ALT* Alanine transaminase, *ALK* alkaline phosphatase, *ICG* Indocyanine green, *PT* Prothrombin time, *PTT* Partial Thromboplastin Time, *RYGB* Roux-en-Y gastric bypass

^a^The gold standard histological diagnosis for significant fatty liver, including nonalcoholic steatohepatitis (NASH) and NASH plus fibrosis, is defined as steatosis >5% with at least a few balloon cells and lobular inflammation >2 foci/200×

^†^The significance criterion was *P* < 0.006 (i.e., 0.05/8, Bonferroni correction)

^b^Area under the receiver operating characteristic curve, which is a good measure of diagnostic accuracy ranging from 0.50 (chance) to 1.0 (perfect prediction)

**Fig. 2:**
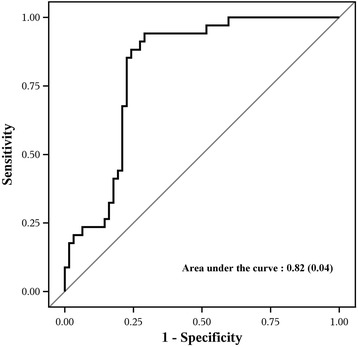
Receiver operating characteristic curves with area under the curve and standard error n parentheses for a multivariable model using all the preoperative liver function tests (except PTT due to a large proportion of missing values) and the ICG *k* value at pre-RYGB to predict significant fatty liver disease. Significant fatty liver, including nonalcoholic steatohepatitis (NASH) and NASH plus fibrosis, is defined as steatosis >5% with at least a few balloon cells and lobular inflammation >2 foci/200×. ICG = indocyanine green, PTT = Partial Thromboplastin Time, RYGB = Roux-en-Y gastric bypass

We also built a multivariable model using all preoperative liver function tests and ICG *k* clearance values to predict NASH from pre-RYGB values. The model had moderate discrimination, reflecting an estimated AUC of 0.82 (95% Cl; 0.73, 0.9, Fig. 2). The estimated sensitivity, specifically, PPV, NPV, and accuracy were 0.86 (95% Cl: 0.75, 0.98), 0.74 (0.63, 0.84), 0.64 (0.51, 0.77), 0.91 (0.83, 0.99), and 0.78 (0.70, 0.86), respectively. The cutpoint is the linear predictor value of −0.918 for which both sensitivity and specificity were maximized. In practice, the multivariable model would be used to calculate a LP value for a patient using the eq. LP = −5.700 + 0.065 x AST + ALT – 0.028 x ALK −0.941 x bilirubin +0.124 x albumin +0.303 x PT + 0.587 x ICG *k* value. The presence of NASH would be predicted by the above formula for a linear predictor value greater than or equal to −0.92.

There was no significant correlation between either ICG *k* clearance and total NAS score (Spearman correlation: −0.03 (97.5% Cl: −0.26, 0.19), *P* = 0.75), or between ICG *k* clearance values and severity of liver disease (Spearman correlation:-0.10 (−0.32, 0.13), *P* = 0.32).

## Discussion

Severely obese patients have substantial histological liver damage by the time they present for bariatric surgery [12]. One-third of patients with liver steatosis typically develop fibrosis without significant clinical manifestations [3], and a substantial proportion of these patients progress towards well-defined NASH with bridging fibrosis over a relatively short period [13]. Consistent with the detrimental effects of morbid obesity on liver histology, two-thirds of our pre-surgical biopsies showed NAFLD with pathologic steatosis. Among these, more than half had clinically important liver damage as defined by NAS scores greater than three or fibrosis.

Previous retrospective and prospective studies have shown that RYGB surgery is associated with successful weight loss and marked histological improvements in steatosis, inflammation, and fibrosis during post-operative follow-up at 12 months [14], while Liu et al. observed resolution of NASH in 60% of 39 patients and rare progression of fibrosis or cirrhosis [9]. Weiner et al. demonstrated complete regression of NAFLD in 83% of 116 patients after a mean postoperative follow-up period of 18.6 months [15]. Similar results were seen in three prospective cohort studies. Klein et al. reported reduced progression of liver fibrosis and inflammation in just 7 patients after 12 postoperative months [16], while de Almeida et al. observed improvements in steatosis, fibrosis, and inflammation in 16 patients after a mean postoperative follow-up period of 23 months [17]. In addition, Furuya et al. demonstrated improved steatosis and fibrosis in 18 patients after 24 months [18]. Two more recent studies have also shown comparable results to those studies above as well as to this study. Caiazzo et al. found that all NAFLD markers improved 5 years after bariatric surgery; however, those markers improved significantly more in severely obese patients following RYGB than after adjustable gastric banding [19]. Lassailly et al. found NASH improved in all patients 1 year after surgery, with better results in those who had gastric bypass rather than gastric banding [20]. Unfortunately, this particular study was conducted over 19 years and both gastric bypass and banding procedures changed over time making their results difficult to interpret.

Only 45 of 105 patients met our criteria for follow-up liver biopsy (NAS score > 3 or fibrosis) and stable weight loss. Although the same NIH-based NAFLD criteria was used as in prior investigations, we determined histological change (non-dichotomous data in rank order) to estimate the mean change from pre- to post-RYGB surgery. There were clinically important and statistically significant histologic improvements from before to after RYGB in steatosis, lobular inflammation, NAS hepatocyte ballooning and fibrosis.

NAFLD describes a broad spectrum of hepatic conditions, which vary historically from simple steatosis to NASH. The diagnosis of NAFLD is often made fortuitously and in the absence of hepatic decompensation is largely asymptomatic. Identification of patients before disease advancement and decompensation may therefore reduce liver-related morbidity and mortality. Junior et al. retrospectively reviewed medical records of 259 RYGB patients who were divided into four groups: normal hepatic biopsy, steatosis, mild NASH, and moderate to severe NASH. They found that aminotransferase and gamma-glutamyl transpeptidase levels as well as fasting glucose were predictors of more advanced stages of NASH.

We also analyzed the correlation between standard biochemical tests of liver function and asymptomatic NAFLD with clinically important liver damage (NAS >3). In the initial single variable model used to estimate correlations between each biochemical liver function test and histological diagnoses, we observed a significant reduction in serum albumin concentrations (*P* < 0.001) as well as significant reductions in AST (*P* = 0.002), and ALT (*P* = 0.02) values in patients with clinically important fatty livers after RYGB. These finding are also consistent with observations by Junior and Nonino-Borges [21]. In addition, reductions in AST and ALT were also associated with more advanced stages of fatty liver including NASH and NASH plus fibrosis.

A second multivariable predictive model was developed using all the preoperative biochemical liver function tests to predict the presence of NASH. The model provided moderate discrimination and therefore, we conclude that based on current clinical measures there are no useful biochemical markers or combinations of clinically available biomarkers for detecting NASH. This observation is consistent with Silverman et al. who also found that biochemical test of liver function correlated poorly with observed hepatic morphological changes after gastric bypass surgery [22].

The ICG clearance test is used for bedside assessment of liver function [23] and has been applied in the perioperative assessment of living liver donors [24]. Interestingly, mean ICG *k* clearance values in our patients with normal/mild fatty liver disease and those with histological fatty livers were similar. The degree of occult fatty liver disease that eventually decreases liver function and alters clearance of ICG remains unknown. Among our patients, ICG clearance was not sufficiently sensitive for detecting clinically important fatty liver (NAS >3) in morbidly obese patients in whom there was no specific clinical or biochemical evidence of liver dysfunction. Thus, this test does not appear to have sufficient sensitivity to discriminate the various progressive stages of fatty liver disease associated with morbid obesity.

Limitations of our study include sample size in the post-op evaluations and potential for measurement error. Of the 45 patients who initially had histological evidence of liver disease and reached the targeted weight-loss or plateau, 25 had follow-up biochemical liver function tests and 15 patients had repeat post-surgery liver biopsies. Since follow-up biochemical and histological tests were only available in a subset of patients we cannot determine the extent to which (self) selection bias may have altered our results. Nevertheless, histologic improvement was remarkable among the patients evaluated, suggesting that gastric bypass surgery at least prevents progression of liver disease, and in most cases reverses existing disease.

Measurement error is also possible in that the degree of liver disease present at the time of RYGB may have been either under- or over-estimated. However, there is no evidence to suggest that liver disease in the morbidly obese is not uniformly distributed throughout the organ. In order to reduce potential error, repeat core needle liver biopsies were obtained from the same lobe of the liver for consistency. Furthermore, a preoperative abdominal ultrasound was performed in every patient and used to guide biopsy location. Thus, there is little reason to believe that sampling error in the biopsies would be anything but random.

## Conclusion

In conclusion, RYGB and weight loss in bariatric patients markedly improved steatosis, lobular inflammation, NAS hepatocyte ballooning and fibrosis. A 70% normalization rate of significant fatty liver disease and stable or reduced severity of fatty liver in the remaining patients suggests that RYGB surgery maybe a promising approach to reverse fatty liver disease, and importantly, a potential future therapeutic option for the treatment and reversal of NASH.
